# Commentary to the article “Transcatheter-based aortic valve replacement vs. isolated surgical aortic valve replacement in 2020”

**DOI:** 10.1007/s00392-022-02111-1

**Published:** 2022-11-23

**Authors:** Kurt Bestehorn

**Affiliations:** TU Dresden, Institute of Clinical Pharmacology, Fetscherstraße 74, 01307 Dresden, Germany

Sirs,

The publication by Gaede et al., published online on April 1st 2022 needs some commentaries:

- According to the methods, the paper is “based on the annual analysis of the data” of the Institute for Quality Assurance and Transparency in Healthcare (IQTIG). That means it is based in reality on published data, the so-called “Bundesauswertung”. The references in this case are the “Bundesauswertung Koronarchirurgie und Eingriffe an Herzklappen: Kathetergestützte isolierte Aortenklappenchirurgie Erfassungsjahr 2020” [federal analysis coronary surgery and intervention on cardiac valves: catheter-based isolated aortic valve surgery year of survey 2020] and “Bundesauswertung Koronarchirurgie und Eingriffe an Herzklappen: Offen-chirurgische isolierte Aortenklappenchirugie, Erfassungsjahr 2020” [federal analysis coronary surgery and intervention on cardiac valves: open surgical isolated aortic valve surgery 2020]. This fact and the source of data are not mentioned in the reference section.
- Baseline characteristics (Table 1) and data of intraprocedural complications (IC) (Table 3) are identical with the “Bundesauswertung” or recalculated in percentages (e.g. overall IC: 6.42%). Interestingly the authors declare 50.5% (*n* = 10,622) as male, according to the original source there are 10,622 females (49.55%).
- As described in the limitations there was no differentiation between transvascular (TV-AVI) and transapical (TA-AVI) possible, which is true for the “Bundesauswertung”. But TV-AVI and TA-AVI patients are very different populations with regard to their risk factor profile and outcome and therefore should be analyzed separately (see Möllmann H. et al. In-hospital outcome of transcatheter vs. surgical aortic valve replacement in patients with aortic valve stenosis: a complete dataset of patients treated in 2013 in Germany, Clin Res Cardiol (2016)). In contrast to the “Bundesauswertung” the original data allow this important differentiation and these data are available. Furthermore, the authors claim “Unfortunately, it is not possible to perform subgroup analyses with regard to the access route”. This is not true: the field 53 of the dataset asks for this information (i.e. endovascular/transseptal/transapical route). A differentiated analysis would have been possible by sending an application to the Federal Joint Committee—for a secondary data analysis. But this was not done as one can see in the list of applications, published by IQTIG. Therefore, the information in the paper is of less value than possible.
- In contrast to the opinion of the authors, that “a different calculation of the risk score models depending on the procedure does not allow a direct comparison of the risk subgroups between iSAVR and TAVI”, this is possible by performing an analysis with the same score for all analysed populations or a matched pair analysis (exact matching) based on the original dataset.
- There is no information about the references for Figs. 1 and 2.
- On page 927 and in Fig. 2 all-cause in-hospital mortality for 2020 is shown for TAVI 2.3% and iSAVR 2,8% with *p* = 0.033 (page 927) and *p* = 0.003 (Fig. 2). What is the value of this information? Both statements are misleading due to the fact that the populations are not comparable: for example, endocarditis is a contraindication for TAVI, which means for reasons of comparability of TAVI vs. iSAVR these patients should be eliminated. There are 631 cases of endocarditis in the iSAVR group (10.3%) vs. 6 in the TAVI group (0.03%). According to the literature the in-hospital mortality rate of endocarditis is about 17–25%, that means possibly 107 or more in-hospital death for the iSAVR population could be associated with endocarditis. Even with a mortality rate of 8% for endocarditis-infected iSAVR cases, the mortality rate of iSAVR cases without endocarditis would be numerically lower than the TAVI mortality. This illustration shows that a comparison of mortality rates between TAVI and iSAVR is of no value for inhomogenous populations as shown in Table 2.
- The decrease of cases e.g. of TAVI by 9.4% (“All procedures—iSAVR, endovascular, and transapical TAVI were performed less frequently in 2020 than in 2019.”) can be explained by the fact that in contrast to 2019 in 2020 there were only patients documented and in the database who are insured by the statutary health insurance—approximtely 90% of the German population! Futhermore, any comparison between the data for 2020 and the previous years must consider these different populations.

## Declarations

## Conflict of interest

The author declares that there is no conflict of interest.

